# Sociocultural adaptation of Chinese international students in the United States and its influencing factors

**DOI:** 10.3389/fpsyg.2025.1607241

**Published:** 2025-07-18

**Authors:** Xiaofang Wei

**Affiliations:** School of Foreign Languages, Lanzhou Jiaotong University, Lanzhou, Gansu, China

**Keywords:** cross-cultural adaptation, Chinese international students, sociocultural adaptation, interpersonal adaptation, environmental adaptation, cultural adaptation, influencing factors

## Abstract

As the number of international Chinese students studying in the US continues to rise and cross-cultural adaptation stress and difficulties they experience may hinder their adaptation culturally, socially, emotionally and academically, understanding their sociocultural adaptation challenges in the host culture becomes increasingly important. The research investigated the cross-cultural adjustment of Chinese students in the US, focusing on the sociocultural adaptation aspect and its influencing factors. A convenience sample of 199 Mainland Chinese students from Kentucky University completed questionnaires assessing sociocultural difficulties (using Sociocultural Adjustment Scale) and psychological well-being (measured by Satisfaction with Life Scale). Factor analysis revealed that sociocultural adaption could be categorized into three sub-scales: interpersonal adaptation, environmental adaptation and cultural adaptation. Among key demographic variables (duration in the US, educational level, and language proficiency) and situational variables (psychological adaptation and social support from host nationals), the multiple regression analyses revealed social support exerts the greatest influence on sociocultural adaptation, while education level has the negative impact on sociocultural adaptation. Language proficiency and social support from host nationals are the strong positive predictors of interpersonal adaptation. Length of residence and psychological adaptation drive environmental adaptation and cultural adaptation most. Limitations of this study, along with possible explanations for the findings, were also discussed.

## Introduction

In the era of globalization, an increasing number of students are opting to pursue higher education abroad in order to enhance their global competencies, facilitate personal development, and boost potential career prospects. Nevertheless, acculturative transitions can be challenging and stressful for international students (Rienties and Tempelaar, 2013) and they may experience acculturative stress and difficulties during their cross-cultural adaptation period (Smith and Khawaja, 2011; Tang and Zhang, 2023), since they need to acquire new life skills to operate effectively in an unfamiliar and challenging cultural environment, to resolve tensions between different cultural perspectives and worldviews and to adapt culturally, socially, emotionally and academically (Bethel et al., 2020; Ward and Szabó, 2019). There is a remarkable surge in the publication of research on international students’ intercultural adaptation, mainly focusing on the process of their adjustment and acculturation, and their experiences and challenges in intercultural adaptation. Notably, this upward trend become particularly pronounced after 2007, underscoring the escalating significance of this field of study (Tang and Zhang, 2023).

According to the Open Doors 2024 Report on International Educational Exchange, China ranks second among all source countries and the number of Chinese students in the United States was 277,398 during the 2023/2024 academic year. As Chinese international students are one of the largest sources of international students in the United States, they face stress and concerns in academic, sociocultural, and personal aspects (Yan and Berliner, 2011) and their cross-cultural adaptation gains researcher’s attention since Chinese students experienced the lowest levels of adjustment and were identified the least adjusted group among all international students because of their prominent levels of stress, neuroticism and perceived cultural distance between mainstream and immigrant culture (Forbush and Foucault-Welles, 2016; Galchenko and van de Vijver, 2007).

Sociocultural adaptation refers to an individual’s effective ability to integrate into a new society and to handle daily intercultural tasks. Given that the sociocultural adaptation is a significant determinant affecting international students’ cross-cultural adjustment (Berry, 1997; Ward and Geeraert, 2016) and it has a significantly positive correlation with academic adaptation and psychological adaptation of international students (Sheng et al., 2022), exploring sociocultural adaptation of Chinese international students in the United States and its influential factors can offer a more holistic comprehension of these students’ experiences within the host nation and make their stay more rewarding.

## Sociocultural adaptation of international students

### Two domains of cross-cultural adaptation: sociocultural adaptation and psychological adaptation

According to Berry (2005), Cross-cultural adaptation is conceptually defined as a complex, multifaceted process that encompasses both cultural and psychological transformations. These changes ensue from the sustained contacts among culturally diverse groups. Ward and her colleagues proposed that cross-cultural adaptation can be categorized into two distinct domains: sociocultural adaptation and psychological adaptation (Ward and Kennedy, 1999; Ward et al., 1998). This theoretical framework has brought the acculturation concept to a functional area of research, enabling researchers to systematically explore the multifaceted nature of acculturation processes. The sociocultural adaptation has been be defined as the process of adapting themselves to the hosting community and functioning successfully in the new environment (Ward and Fischer, 2008), which involves developing social and cultural skills, respecting the beliefs, values, and norms of the new culture, and acquiring sufficient communication skills to communicate with the host community effectively (Castro and Murray, 2010). Accordingly, the sociocultural adaptation involves the development of social and cultural ability to fit in the host culture or interact effectively with local members, which can be predicted by variables concerned with facilitating cultural learning and acquiring social skills in the host culture (Oguri and Gudykunst, 2002). The addition of a cognitive dimension to the behavior-based sociocultural adaptation has been explored in recent researches. Kelley and Meyers (1995) identified perceptual acuity is a major component of cross-cultural adaptability. Ward and Kennedy (1999) suggested sociocultural adaption might split into cognitive and behavioral sub-scales: Cultural Empathy and Relatedness and Impersonal Endeavors and Perils. The former relates to cognitive and interpersonal relationship items and the latter concerns the management of interpersonal interactions and awkward situations.

Psychological adaptation has been conceptualized through the affective dimension as the sense of happiness and satisfaction (Ward et al., 1998), as well as the degree to which an individual subjectively experiences happiness and comfort while residing in the host country (Ward and Rana-Deuba, 1999). Psychologically adjusted individuals are characterized by psychological well-being or satisfaction in their host culture (Oguri and Gudykunst, 2002), a state influenced by emotional states, cognitive perceptions, and personal trait variables. When entering into the host-culture, international students may experience stressful encounters and challenges ranging from practical, cultural, social and academic difficulties (Jung et al., 2007; Olivas and Li, 2006). The stressful encounters and challenges experienced by international students often trigger feelings of uneasiness, insecurity, and loss and may cause acculturative stress and variety of negative outcomes for international students, including depression, anxiety, and somatic symptoms (Yakunina et al., 2013).

Empirical studies suggests although psychological and sociocultural adjustment are conceptually and empirically distinct, they are positively correlated. However, these two forms of adjustment follow somewhat different developmental trajectories over time and are influenced by different sets of variables (Berry, 2005; Demes and Geeraert, 2014; Ward et al., 1998). The sociocultural adaptation pertains to an individual’s ability to fit in the host environment effectively, which is most comprehensively understood within a social skills or culture learning paradigm (Brisset et al., 2010; Ward and Kennedy, 1999). In contrast, psychological adjustment, primarily associated with feelings like satisfaction and well-being (Brisset et al., 2010), is significantly influenced by variables such as personality, coping styles, and support. Given the conceptual and empirical distinctions between these domains, different assessment scales are employed to measure psychological and sociocultural adaptation of individuals within the host culture.

### Cross-cultural adaptation of Chinese international students and its influential factors

International students are a special group who live in a foreign country pursuing an educational goal, with their own cross-cultural adaptation difficulties and challenges. Unlike immigrants, International students who may consider their stay in the US to be temporary, may try to balance between maintaining their traditional roles, adjusting to the new culture (Lee and Çiftçi, 2014) as well as achieving their academic success which is of importance to them (Young et al., 2013). This focus on academic progress and cultural adaptation presents unique challenges and opportunities for international students as they navigate their educational journey in a foreign land.

Sociocultural adaptation which is defined in terms of behavioral competence refers to the ability to acquire knowledge and skills to cope with and handle daily life affairs. It can be best explained within a social skills and cultural learning paradigm (Ward and Kennedy, 1999). It involves behavioral responses in the process of cross-cultural adaptation and the successful handling of cross-cultural situations. For international students, sociocultural adaptation is an important factor that influences international students’ cross-cultural adjustment. They need to acquire new life skills to operate effectively in an unfamiliar and challenging cultural environment in order to resolve tensions between different cultural perspectives and worldviews and to adapt culturally, socially and academically (Bethel et al., 2020; Ward and Szabó, 2019). Findings from the longitudinal study conducted by Cemalcilar and Falbo (2008) even indicated that positive acculturation correlated with sociocultural adaptation, as opposed to having a significant association with psychological well-being or academic adaptation. For international students, successful sociocultural adaptation equips them with the resilience to overcome challenges and fosters their mental well-being. By building friendships and communicating with people, international students could significantly improve their general well-being (Neri and Ville, 2008). Furthermore, Sociocultural adaptation has been proven to be significantly positively associated with academic adaptation (Sheng et al., 2022) and students who are able to fit in the sociocultural aspects of the academic setting, such as understanding the implicit rules of academic discourse and interacting well with teachers and peer students, are more likely to perform well academically. Frequency of social interactions between overseas and domestic students promote mutual understanding and break down cultural barriers, thus enhancing cultural awareness and cultivating global citizens (Bethel et al., 2020). The sociocultural adaptation of international students involves the acquisition of behavioral skills and cultural knowledge to navigate daily life in a host culture, exerting profound influences on their mental well-being, academic achievement, global competence, and future professional trajectories.

Sociocultural adaptation conceptualizes cross-cultural adaptation in terms of behavioral competence and it is fundamentally shaped by factors on cultural learning and social skills acquiring (Ward and Kennedy, 1999). These factors include some key demographic variables, like age, gender, ethnicity, educational level, length of residence in the host culture (Ward and Kennedy, 1999; Alharbi and Smith, 2018; Yerken et al., 2022). Language proficiency also acts as a significant predictor of sociocultural adaptation. Mastery of the host language enhances their participation in host-culture social activities, reduces barriers to academic engagement, thereby mitigating sociocultural stressors (Ward and Kennedy, 1999; Alharbi and Smith, 2018; Yerken et al., 2022; Bethel et al., 2020). Several studies have highlighted the advantages of social support in facilitating adjustment, promoting academic achievement and managing life stressors (Lee and Çiftçi, 2014; Alharbi and Smith, 2018; Bethel et al., 2020; Yerken et al., 2022; Luo et al., 2024). Low levels of intercultural engagement and identification with host nationals has been identified as a key challenge in international education (Bethel et al., 2020; Ward and Kennedy, 1999). A cross-cultural comparison study revealed that European students encounter fewer difficulties in making friends from the host country and mastering the host country’s language than Asian students (Alharbi and Smith, 2018), hence less adjusted. Psychological adaptation is also another significant factor that shapes sociocultural adaptation. Positive mental health motivates international students to adopt more positive problem-focused coping strategies and encourage them to engage with more cultural activities, thus accelerating sociocultural learning (Bethel et al., 2020; Ward and Rana-Deuba, 1999). Evidently, the relationship between the two dimensions of adjustment is expected to be positive although its strength varies in different studies (Hirai et al., 2015; Ward et al., 1998).

Based on our literature review, no sampling-based quantitative research has been conducted to systematically examine the sub-dimensions of sociocultural adaptation among international students from China. Given the growing population of Chinese international students in the United States and the significant challenges they encounter in sociocultural adaptation to the host culture, investigating their sociocultural adaptation process and its sub-dimensions holds substantial academic and practical significance. Additionally, while previous studies have predominantly examined the impact of various factors on sociocultural adaptation as a whole, this research will investigate the influences of these factors not only on the overall sociocultural adaptation of Chinese international students but also on their specific sub-dimensions.

### Purpose of study

This study aims to investigate the underlying sub-dimensions of sociocultural adaptation of Chinese international students and to explore how key demographic variables (duration in the US, educational level, and language proficiency) and situational variables (psychological adaptation and social support from host nationals) affect sociocultural adaptation and its sub-dimensions adaptation. The exploration of the sociocultural adaptation process of Chinese international students in the US would aid the understanding of their sociocultural adaptation patterns, improve their sociocultural as well as psychological adaptation, and carry educational implications for cross-cultural adaptation training both prior to departure and during the staying in the host culture. We formulated several hypotheses regarding the anticipated association between sociocultural adaptation and different variables.

Research Question 1: Does sociocultural adaptation have sub-dimensions? If so, how are these sub-dimensions structured and interrelated?

Research Question 2: How do the demographic variables of Chinese international students in the US (such as length of residence, educational level and language proficiency) affect their degree of sociocultural adaptation?

Research Question 3: How does the psychological adaptation of Chinese international students in the US affect their degree of sociocultural adaptation?

Research Question 4: How does social support from host nationals affect their degree of sociocultural adaptation?

## Methods

### Subjects

Participants were required to be Mainland Chinese international students enrolled in an undergraduate or postgraduate program. A total of 199 Mainland Chinese sojourning students from the Kentucky University in the US participated in this study. The sample was obtained through convenience sampling. The questionnaire was collected in two ways. Firstly, the electronic questionnaires were distributed to Mainland Chinese students with the help of Education College of Kentucky University and data were collected through the questionnaire website.^1^ Secondly, paper questionnaires were mailed to Mainland Chinese students who lived in the university hostel with the help of several Mainland Chinese students. A total of 216 questionnaires were collected, 199 of them were valid and 7 participants did not complete the full questionnaire, including 83 paper questionnaires and 116 electronic questionnaires. The sample consisted of 88 males (44.2%) and 111 females (55.8%). Among them, 78 students were between 18 and 25 years (39.2%), 67 students were between 26 and 34 years (33.7%) and 54 students were 35 years or above (27.1%). Regarding their education background, 46 students (23.1%) were undergraduates, 49 students (24.6%) were master’s students and 104 students (52.3%) were doctoral students. Their length of residence in the US ranges from 1 month to 6 years, with 61 students (30.7%) staying there for 1 to 3 months, 57 students (28.6%) staying there for 3 to 12 months and 81 (40.7%) students staying there for 1 year and above.

### Measures

#### Demographics

The questionnaire included demographic variables such as gender, age, educational attainment, duration of residence in the United States, and English language proficiency. Given the importance of language proficiency in cross-cultural adaptation, self-reported language proficiency measures were utilized to evaluate the language proficiency of international students. English language proficiency was assessed by the question “How would you rate your overall English language ability?,” and the responses were rated on a five-point Likert scale, where a score of “1” denoted “very poor” proficiency, and a score of “5” represented “very good” proficiency.

#### Sociocultural adaptation

The present study adopted the Sociocultural Adjustment Scale (SCAS) to assess sociocultural adaptation of Chinese international students. Based on Furnham and Bochner's (1982) study, Searle and Ward (1990) first applied the SCAS to investigate cross-cultural adjustment of Malaysian and Singaporean students studying in New Zealand. The reliability and validity of the SCAS have been repeatedly demonstrated, and it has been adopted by a number of studies to assess the cross-cultural adaptation and intercultural competence of respondents (Berry and Sabatier, 2010; Saricoban and Oz, 2014; Savicki, 2008; Ward et al., 1998; Ward and Kennedy, 1999; Ward and Rana-Deuba, 1999).

The SCAS (Ward and Kennedy, 1999) is a 28-item scale that assesses the amount of difficulty experienced in various sociocultural situations. Ward and Kennedy (1999) suggested the SCAS scale has a flexible structure that can be adapted to suit the specific characteristics of the research population. Based on the frontier study and feedback of Chinese students, the SCAS was adjusted to remove five items that are irrelevant for Chinese students to make it more understandable and user-friendly. The SCAS used in the study was composed of 23 items, investigating the social and cultural skills required to manage everyday matters in the host culture. Chinese international students were required to rate their existing intercultural adaptation difficulties on five-point Likert scales in five levels: (1) no difficulty, (2) slight difficulty, (3) medium difficulty, (4) very difficult, and (5) extreme difficulty and higher scores indicate greater difficulty in adapting to the new culture. The scale involves categories such as making friends with local people, socializing with authority figures, interacting with the opposite sex, coping with unsatisfactory service, social habits, pace of life, diet, accommodation, climate, transportation, knowing the way, language, way of thinking, conversation style, values, cultural perspective, political system and religious belief. Before data analysis, the reliability analysis of the scale was carried out (Cronbach’s a = 0.900), indicating a high reliability of the scale.

#### Psychological adaptation

Since psychological adaptation refers to feelings of well-being or satisfaction and can be understood within a stress and coping framework, Satisfaction with Life Scale (SWLS; Diener et al., 1985) was used to measure psychological adaptation of Chinese international students in the research. It was proved that the SWLS has favorable psychometric properties with high internal consistency and high reliability (Diener et al., 1985). The SWLS is a five-item questionnaire using a 7-point Likert scale (from 1 = strongly disagree to 7 = strongly agree) to measure individual’s overall life satisfaction. According to the responses to statements such as “in most ways my life is close to my ideal” and “the conditions of my life are excellent,” the overall satisfaction with life of respondents has been measured and higher scores indicates greater life satisfaction. The SWLS can be viewed as a measure of psychological adaptation, since the scale demonstrated moderately strong criterion validity with several measures of psychological well-being (Savicki, 2008). The Cronbach’s alpha coefficient was 0.886, which suggested relatively high internal consistency.

#### Social support

The study utilized Multi-Dimensional Support Scale (MDSS; Winefield et al., 1992) to assess social support Chinese international students received from host friends (American friends). Subjects rated the frequency of receiving support from host friends in the last month on a 4-point scale ranging from never (1) to always (4). The researching questions included the following 5 questions “(1) How often did your host international friends really listen to you when you talked about your concerns or problems? (2) How often did you feel that they were really trying to understand your problems? (3) How often did they try to take your mind off your problems by telling jokes or chattering about other things? (4)How often did they help you in practical ways, like doing things for you or lending you money? (5) How often did they answer your questions or give you advice about how to solve your problems?” Scores are averaged across the five items, with higher scores indicating stronger perceived social support. The scale exhibits strong internal reliability (Cronbach’s alpha = 0.848), providing empirical support for its use in research.

## Results

### Overall distribution of sociocultural adaptation categories

The sociocultural adaptation difficulties experienced by Chinese international students in the United States were assessed across 23 categories, with mean values varying significantly. Notably, some categories presented more pronounced sociocultural adaptation challenges than others. The highest difficulty was observed in the category “Dealing with Americans who is unpleasant” (M = 2.69), while the lowest was found in “Coping with the differences in environmental protection between China and the United States” (M = 1.35). These findings highlight that the adaptation challenges faced by Chinese international students are not randomly distributed, with certain aspects of sociocultural adaptation being more salient than others.

### Dimensions of sociocultural adaptation

Recent studies have explored the incorporation of additional dimensions into the traditional behavior-based Sociocultural Adaptation Scale (SCAS; Ward and Kennedy, 1999). Based on the frontier study and feedback from Chinese students, an 23-item version of the SCAS was employed to assess the sociocultural adaptation difficulties faced by Chinese students in the United States. The SCAS was examined by factor analysis to refine its measurement capacities.

A structural validity analysis should be performed before a factor analysis. From the KMO and Bartlett tests in Table 1, it can be seen that the KMO value of 0.873 suggested a strong degree of common variance among the variables, which passed the Bartlett’s test of Sphericity test with a significant *p*-value, indicating that the original data of the social adaptation scale had good structural validity and was suitable for subsequent factor analysis. Overall, these results indicated that factor analysis can proceed with confidence, suggesting that the 23-item SCAS was likely to have a valid and meaningful factor structure.

**Table 1:** KMO and Bartlett’s test.

Measure	Statistic	Value
KMO measure of sampling adequacy		0.873
Bartlett’s test of sphericity	Approx. chi-square	2202.867
	df	253
	Sig.	<0.001

The factors were extracted by Principal Component Analysis (PCA), and the factor whose eigenvalue was greater than 1 was selected. The factor is rotated by Varimax with Kaiser Normalization, and rotation converged in 5 iterations. To ensure robustness, measurement items fewer than two factor loadings, factor loadings below 0.5, or items that cross-loaded on multiple factors were excluded. The factor analysis resulted in three distinct factors, explaining a cumulative 63.17% of the variance, which indicates that the three factors were well representative of the data. All items had factor loading greater than 0.5 and were clearly associated with their respective factors. The factor analysis result for the sociocultural adjustment scale, containing 18 items, was presented in Table 2.

**Table 2:** Factor analysis of the SCAS.

Categories	Factor		
	1	2	3
Y20 express yourself clearly	0.842		
Y2 dealing with people in authority	0.822		
Y1 making friends with local people	0.804		
Y3 relating to numbers of the opposite sex	0.777		
Y21making yourself understood	0.772		
Y23 social contact: daily greetings and interactions	0.758		
Y19 getting used to different communication styles and topics	0.704		
Y9getting used to the pace of life		0.790	
Y6 dealing with the climate		0.778	
Y7 adapting to local accommodation		0.715	
Y10 finding your way around		0.681	
Y8 using the local transport system		0.671	
Y5 finding food you enjoy		0.669	
Y14 understanding the local value system			0.778
Y16 understanding cultural differences			0.775
Y18 understanding the local political system			0.757
Y15 seeing things from the locals’ point of view			0.727
Y17 understanding local religious system			0.683
Total	4.657	3.466	3.248
% of variance	25.874	19.256	18.044
Cumulative %	25.874	45.130	63.174

Extraction method: principal component analysis. rotation method: varimax with Kaiser normalization.

The result of factor analysis indicated that items Y20, Y2, Y1, Y3, Y21, Y23 and Y19 all loaded onto the same factor (Factor I). All items in factor I had a strong load (> 0.7), and the items included were related to dealing with interpersonal relationships and communication in daily life in the host culture, so factor I was labeled as interpersonal adaptation which reflects the aspect of communicative adaptation which includes interpersonal relationships like making friends with local people, dealing with people in authority or of opposite sex, and communication like expressing yourself clearly, making yourself understood and social contact.

Factor II included 6 items, Y9, Y6, Y7, Y10, Y8 and Y5, whose load values were all above 0.6. and this factor was labeled as environmental adaptation, which mainly involved the adaptation to the living environment in host culture. Environmental adaptation included adaptation to local climate, local food, transportation system, pace of life and finding your way.

Based on the analysis of factor III, Y14, Y16, Y18, Y15 and Y17 belonged to the same factor, which was labeled as cultural adaptation, including the understanding of local culture and cognition in cross-cultural activities, involving five aspects such as understanding the local values, cultural differences, perspectives, and political and religious system.

The sociocultural adaptation reliability test was shown in Table 3. It can be seen that the overall reliability of the sociocultural adaptation scale was 0.911, indicating excellent internal consistency for a scale as a whole. The reliability of the sub-dimensions of interpersonal adaptation, environmental adaptation and cultural adaptation were 0.917, 0.835 and 0.849 respectively, greater than the standard of 0.7, which demonstrated high internal reliability for the items in these 3 factors. These high Cronbach’s Alpha values indicated that the Sociocultural Adaptation Scale is reliable and consistently measures the different aspects of the sociocultural adaptation.

**Table 3:** Reliability statistics of SCAS.

SCAS (18 items)	Factors	Cronbach’s alpha	Items
Cronbach’s alpha = 0.911	Factor 1: Interpersonal adaptation	0.917	7
	Factor 2: Environmental adaptation	0.835	6
	Factor 3: Cultural adaptation	0.849	5

Through confirmatory factor analysis (CFA) in AMOS, all absolute fit indices, incremental fit indices, and parsimony-adjusted indices met the judgment criteria (see Table 4). The table displayed the fit results of the factor analysis model for sociocultural adaptation. By assessing multiple fit indices, the model receives an overall evaluation of “very good” across all indices, indicating the model’s appropriateness for the underlying factors of sociocultural adaptability. The model demonstrates a high degree of fit, further suggesting that the sociocultural adaptability scale employed in this study exhibits good validity and that the three extracted common factors are reliable.

**Table 4:** Confirmatory factor analysis (CFA) model fit summary for sociocultural adaptation scale.

Index	Absolute fit indices				Incremental fit indices			Parsimony-adjusted indices	
	χ^2^/df	GFI	RMR	RMSEA	NFI	TLI	CFI	PGFI	PNFI
Threshold	<3	>0.9	<0.08	<0.08	>0.9	>0.9	>0.9	>0.5	>0.5
Fit Results	1.487	0.904	0.024	0.050	0.902	0.960	0.965	0.698	0.778
Fit Evaluation	Excellent				Excellent			Excellent	

### Analysis of influencing factors of sociocultural adaptation

This study employed multiple regression analysis to examine the determinants of sociocultural adaptation and its dimensional structure (interpersonal, environmental, and cultural adaptation) among Chinese international students. The model incorporated five predictors: length of residence, education level, language proficiency, psychological adaptation, and social support from host nationals. Table 5 presented the results of a multiple regression analysis, which examined how various factors influenced sociocultural adaptation and its three sub-dimensions: interpersonal adaptation, environmental adaptation, and cultural adaptation. The model includes standardized coefficients (*β*), standard errors (in parentheses), and significance levels (*p*-values), as well as overall fit statistics (R, R^2^, F-test). All models are statistically significant (*p* < 0.001), validating the utility of the models.

**Table 5:** Multiple regression analysis of factors influencing sociocultural adaptation.

Influencing factor	Sociocultural adaptation and its three dimensions (dependent variables)			
	Sociocultural adaptation	Sub-dimension 1: interpersonal adaptation	Sub-dimension2: environmental adaptation	Sub-dimension 3: cultural adaptation
Length of residence	0.128*(0.013)	0.077(0.021)	0.185*(0.019)	0.161*(0.021)
Education level	−0.144*(0.028)	−0.112*(0.044)	−0.159*(0.039)	−0.083(0.043)
Language proficiency	0.234***(0.028)	0.329***(0.045)	−0.020(0.040)	0.131(0.044)
Psychological adaptation	0.223***(0.021)	0.167**(0.033)	0.208**(0.029)	0.160*(0.032)
Social support from host nationals	0.260***(0.049)	0.315***(0.079)	0.056(0.070)	0.060(0.077)
*R*	0.642	0.697	0.360	0.369
*R^2^*	*0.412*	*0.486*	*0.130*	*0.136*
*F*	27.090***	36.564***	5.754***	6.079***

Standardized coefficients (β) and standard errors (in parentheses) are reported. *p < 0.05, **p < 0.01, ***p < 0.001 (two-tailed test).

As outlined in Table 5, the multiple regression analysis revealed the model explains 41.2% of the variance in sociocultural adaptation (R^2^ = 0.412) and all predictors collectively produce a statistically significant effect (*F* = 27.090, *p* < 0.001). Among the factors influencing sociocultural adaptation, social support from host nationals (*β* = 0.260, *p* < 0.001), language proficiency (*β* = 0.234, *p* < 0.001), and psychological adaptation (*β* = 0.223, *p* < 0.001) have the strong positive effects, followed by length of residence (*β* = 0.128, *p* < 0.05), while education level exhibits a significant negative association (*β* = −0.144, *p* < 0.05). The result highlights social support from host nationals emerges as the strongest predictor of sociocultural adaptation, while education level has the negative impact.

The multiple regression analysis for interpersonal adaptation indicated the model explains 48.6% of the variance (R^2^ = 0.486), with a strong overall significance (*F* = 36.564, *p* < 0.001). Language proficiency (*β* = 0.329, *p* < 0.001) and social support from host nationals (*β* = 0.315, *p* < 0.001) are the two strongest positive predictors, followed by psychological adaptation (*β* = 0.167, *p* < 0.01), while education level has a significant negative effect (*β* = −0.112, *p* < 0.05). The result emphasizes the critical role of language competence, social support, and mental well-being in fostering social relationships, while reveals potential barriers to interpersonal integration linked to educational background.

The multiple regression analysis for environmental adaptation revealed that length of residence (*β* = 0.185, *p* < 0.05) and psychological adaptation (*β* = 0.208, *p* < 0.01) are significant positive predictors of environmental adaptation, indicating that longer stays and better mental well-being enhance adaptation to environment. In contrast, education level has a significant negative effect (*β* = −0.159, *p* < 0.05), potentially reflecting higher education level is associated with poorer environmental adaptation. Language proficiency and social support from host nationals are non-significant predictors (*p* > 0.05), and they have limited direct impact on environmental adaptation. The model explains only 13.0% of variance (R^2^ = 0.130), though the overall model is statistically significant (*F* = 5.754, *p* < 0.001). The result highlights the role of length of residence and psychological factors in environmental adaptation while indicates that other unmeasured variables may also influence it significantly.

The multiple regression analysis for cultural adaptation demonstrated that length of residence (*β* = 0.161, *p* < 0.05) and psychological adaptation (*β* = 0.160, *p* < 0.05) are significant positive predictors of this sub-dimensions, indicating that longer exposure to the host culture and better mental well-being facilitate cultural assimilation while education attainment presents a non-significant negative impact (*β* = −0.083, *p* > 0.05), suggesting no strong link between higher education and better cultural adaptation. Language proficiency (*β* = 0.131) and social support from host nationals (*β* = 0.060) also have non-significant effects with limited direct influence on internalizing cultural norms. The model explains 13.6% of variance (R^2^ = 0.136), though the overall fit is statistically significant (*F* = 6.079, *p* < 0.001). The result highlights the role of time and psychological factors in cultural adaptation but it also suggests other unmeasured elements may play a more substantial role in this sub-dimension.

The multiple regression analyses across sociocultural adaptation and its three dimensions show social support exerts the greatest influence on sociocultural adaptation, while education level has the negative impact on sociocultural adaptation. Language proficiency and social support from host nationals are the strong positive predictors of interpersonal adaptation. Length of residence and psychological adaptation drive environmental adaptation and cultural adaptation most. All the regression analysis results are statistically significant, although the suggested variables exhibit lower explanatory power for environmental and cultural adaptation, indicating the presence of other unmeasured significant factors influencing these two sub-dimensions.

## Discussion

The study sought to enhance the overall understanding of the adaptation experiences of Chinese students in the United States, focusing on two primary aspects: the sociocultural adaptation of Chinese students in the United States and influential factors on sociocultural adaptation. Ward and Kennedy (1999) suggested items of sociocultural adaption scale might split into two sub-scales through factor analysis, Cultural Empathy and Relatedness and Impersonal Endeavors and Perils. However, through factor analysis of the data in this research, findings suggested the categories in sociocultural adaption would fall into three sub-dimension. The first sub-dimension related to interpersonal adaptation including items dealing with interpersonal relationships and communication in daily life in the host culture. International students need to build new social networks to provide social resources and help them adapt to the new social and academic environment, reducing loneliness and the risk of depression (Sawir et al., 2008; Sadewo et al., 2020). If international students interact with host nationals on a regular daily basis, they may undergo reduced social difficulties (Ward and Kennedy, 1999) and their communication competency and adaptation skills, as well as social connectedness, would be enhanced (Tang and Zhang, 2023). The second sub-dimension was named environmental adaptation and composed of items relating to the adaptation to the living environment in the host country. It was evident that international students in the United States encountered significant challenges due to the unfamiliar environment they were living. It was suggested that practical problems in daily living, such as securing appropriate accommodation would be stressful and challenging (Bradley, 2000; Sawir et al., 2008; Bethel et al., 2020). The third dimension was named cultural adaptation, including the items involving understanding the local values, cultural differences, perspectives, and political and religious system. The tendency to accept and value other cultures would be included as a key component of cross-cultural adaptation (Ward and Kennedy, 1999). While these findings are preliminary, this line provides valuable insights into understanding Chinese sociocultural adaptation through the three sub-dimensions, which merits further exploration.

The multiple regression results indicate that the five independent variables collectively account for 41.2% of the variance in overall sociocultural adaptation. Length of residence, educational attainment, English proficiency, psychological adaptation, and social support from host nationals each significantly influence the sociocultural adaptation of Chinese international individuals, yet their influence varies both in magnitude and in the specific dimensions of adaptation they affect.

Length of Residence: Length of residence and sociocultural adaptation correlated with each other significantly and the more time the student had lived in the host culture, the less sociocultural adaptation difficulty they would experience (Kuo and Roysircar, 2004; Wang et al., 2018; Ward and Kennedy, 1999; Wilton and Constantine, 2003). Regression results in this study also indicated a positive relationship between the length of residency in the U. S. and sociocultural adaptation. However, among three sub-dimensions of sociocultural adaptation, this variable was found to be positively associated with environmental adaptation (*p* < 0.05) and cultural adaptation (*p* < 0.05), but not with interpersonal adaptation. This suggests prolonged stay enhances familiarity with local environments and cultural practices, whereas building relationships with host nationals requires active social engagement rather than mere duration of stay. Wilton and Constantine (2003) mentioned international students of longer residence tended to report lower levels of difficulties in adjusting to local cultural norms and more likely to establish social support networks. Longer residence period in a host country was associated with deeper cultural immersion and greater familiarity with the host culture (Young et al., 2013), which could be reflected in higher levels of intercultural awareness and behavioral adaptability, leading to better adaptation in environmental and cultural adaptation. The limited social interactions between international and domestic students and the low occurrence of intercultural friendships were described as a serious issue during their stay in host culture (Quinton, 2020; Smart et al., 2000; Smith and Khawaja, 2011), especially for East Asian students in Western countries (Rienties and Tempelaar, 2013; Bethel et al., 2020) and longer residence in the host country can not alleviate their interpersonal adaptation difficulties.

Education level: Education level exhibits significant negative effects on sociocultural, interpersonal, and environmental adaptation but not on cultural adaptation. However, compared to other factors, educational level exerts the weakest influence on overall sociocultural adaptation and its sub-dimensions. In other words, individuals with higher educational level tend to exhibit poorer adaptation in interpersonal and environmental contexts. Higher levels of research pressure and more demanding academic goals perhaps put international students of higher education levels into extra acculturative difficulties. Gebregergis (2018) similarly highlighted in his discussion on the cross-cultural adaptation that Master and Doctorate international students faced greater academic stress and work-related pressure, than the Bachelors, leading to more pronounced adaptation challenges during their overseas experiences. Higher education levels may involve another factor age that contributes to maladaptation. Some researches indicated that younger individuals reported lower level of acculturative stress because they are more flexible and open to integrating with the mainstream culture (Berry, 1997; Gebregergis, 2018). Furthermore, international students of different education levels tend to adopt varied cross-cultural coping strategies. For highly educated populations, formal education systems serve as institutionalized channels for the deep internalization of native cultural norms which may hinder adaptation to divergent cultural frameworks. International students of higher levels who strongly identify with their Chinese identity may face more challenges in adapting to host social norms. When individuals were holding on to their original culture, and at the same time wish to avoid host culture, then the Separation strategies was adopted, leading to negative sociocultural and psychological adaptation outcomes (Berry, 1997). The moderating role of education in sociocultural adaptation, interpersonal and environmental dimensions may be attributed to factors such as age, higher academic goals, research pressure, and adopting different cross-cultural coping strategies, however, the influence is very limited.

Language Proficiency: Among various stressors, language competence has been widely recognized as a crucial indicator for measuring cross-cultural adaptation stress. Based on the regression analysis, language proficiency significantly influences sociocultural adaptation and interpersonal adaptation (*p* < 0.001), highlighting language proficiency is a key enabler for social interaction and emotional connection. The significance of host language proficiency for foreigners is well documented and researches highlighted its role in facilitating social interactions and reducing acculturative stress (Sheng et al., 2022; Luo et al., 2024). The research by Gebregergis (2018) indicated that Language barriers, especially oral communication, was perceived as a major challenge for many international students. Host language proficiency is consistently identified as key predictor of academic and interpersonal adaptation of international students. Yeh and Inose (2003) revealed language proficiency also facilitated everyday communication, making it easier for international students to make friends and seek help. However, its lack of impact on environmental and cultural sub-dimensions suggests these domains rely more on practical experience or deep cultural immersion, where language is a baseline rather than a driver.

Psychological Adaptation: Psychological adaptation and sociocultural adaptation are two dimensions of cross-cultural adaptation. While these two dimensions are distinct, they are significantly correlated with each other. The higher the life satisfaction of international students, the better their sociocultural adaptation, and the lower their perceived adaptation difficulties. (Berry, 2005; Demes and Geeraert, 2014; Ward and Kennedy, 1999; Zheng and Ishii, 2023). Regression analysis also proved that psychological adaptation is positively correlated with sociocultural adaptation and all its three sub-dimensions(with varying *p*-values: *p* < 0.001 for total, *p* < 0.01 for interpersonal and environmental, *p* < 0.05 for cultural). Compared to other factors, psychological adaptation was the only variable that significantly affects sociocultural adaptation and all three sub-dimensions. International students with more social personality, high level of social belonging, social support network satisfaction and social connectedness which are core components of psychological adaptation tend to create a more profound connection with host nationals (Rivas et al., 2019; Yeh and Inose, 2003). The findings in this study also confirmed that psychological adaptation is a foundational factor for adapting to diverse sociocultural contexts, which influence how individuals perceive and navigate challenges in all dimensions of sociocultural adaptation, including interacting with host nationals, adapting to local environment and appreciating different cultures.

Social Support from Host Nationals: The study indicated social support from host nationals is a significant influencing factor for sociocultural adaptation and interpersonal adaptation sub-dimension (both *p* < 0.001), with standardized coefficients of 0.260 and 0.315, respectively. Among all independent variables, social support from host nationals claims the highest standardized regression coefficient (0.260) for sociocultural adaptation, suggesting that it has a stronger predictive effect on adaptation than other factors. According to previous research, social support from host nationals endows international students with improved communication competence and social skills, which are highly involved with sociocultural adaptation (Geeraert and Demoulin, 2013; Hirai et al., 2015, Luo et al., 2024). It has been further indicated that social support from co-nationals and families contribute to the subjective well-being, whereas social support form host nationals may assist the adaptation to the new cultural environment (Ng et al., 2013). This study demonstrates the same conclusion that greater support from host nationals significantly facilitate sociocultural adaptation. As for interpersonal sub-dimension, the regression result showed social support from host nationals strongly correlates with higher levels of interpersonal adaptation in daily communication. Previous research indicates that Asian students often experience challenges forming relationships with American peers, attributed to differing social norms (Chapdelaine and Alexitch, 2004; Lee and Çiftçi, 2014). Contact with host students can assist in the acquisition of culturally appropriate skills and behaviors (Lee and Çiftçi, 2014), which to some extent can facilitate the interpersonal adaptation within the host culture. Adopting different communication strategies would affect the degree of sociocultural adaptation and the more cross-cultural contact and the higher the satisfaction of contact, the more social support they will get and the less sociocultural difficulties they would encounter (Berry, 2005). However, the factor shows no significant association with environmental adaptation or cultural adaptation (both *p* > 0.05), suggesting that while direct social connections with host nationals effectively enhance sociocultural adaptation and interpersonal communication, they have limited impact on adapting to physical environments or deep cultural norms, implying that environmental and cultural adaptation may rely more on other unmeasured factors rather than direct social support.

The above discussion indicates the key factors influencing sociocultural adaptation include social support from host nationals, language proficiency and psychological adaptation, particularly in interpersonal contexts. Cross-cultural training programs should prioritize language skills, psychological adaptation, and opportunities for meaningful engagement with host communities like community service or cultural exchange events to facilitate international students’ overall sociocultural adaptation. As for three sub-dimensions, language proficiency and social support dominate in interpersonal adaptation, whereas environmental and cultural adaptation rely more on residence duration and psychological adaptation. The adaptation of each sub-dimension can be improved by enhancing its relevant influencing factors.

### Research limitations

While this study offered valuable insights into the sociocultural adaptation of Chinese international students and its influencing factors, it was not without limitations. The relatively small sample size of 199 participants restricted the analyses and it might be difficult to generalize the results to all Chinese international students. Further studies are needed to evaluate the generalization of these findings to other samples of Chinese international students. Additionally, the adaptation process is dynamic and influenced by multiple variables, factors that are identified as causing cross-cultural difficulties may evolve at different phases of the cross-cultural adaptation process. Longitudinal studies are recommended to clarify the adaptation process for Chinese international students and to explore the dynamic relationship between its two dimensions over time. Furthermore, although the model explains 41.2 and 48.6% of variance in overall sociocultural adaptation and interpersonal adaptation sub-dimension, indicating moderate explanatory power, only 13.0 and 13.6% of the variance in environmental adaptation and cultural adaptation, indicating that key influencing factors are missing from the analysis. These unmeasured variables play a significant role in how individuals adapt to physical environments and cultural norms, limiting the model’s predictive power for these two sub-dimensions. Further research is needed to explore other key factors influencing environmental adaptation and cultural adaptation.

## Data Availability

The raw data supporting the conclusions of this article will be made available by the authors, without undue reservation.

## Footnotes

^1^ www.wenjuan.com
